# Dynamic Contrast Enhanced MRI Can Monitor the Very Early Inflammatory Treatment Response upon Intra-Articular Steroid Injection in the Knee Joint: A Case Report with Review of the Literature

**DOI:** 10.1155/2011/578252

**Published:** 2011-03-17

**Authors:** Mikael Boesen, Olga Kubassova, Marco A. Cimmino, Mikkel Østergaard, Peter Taylor, Bente Danneskiold-Samsoe, Henning Bliddal

**Affiliations:** ^1^Department of Radiology, Frederiksberg Hospital, Nordrefasanvej 57, Frederiksberg Hospital 2000 Frederiksberg, Denmark; ^2^Parker Institute, Frederiksbeg Hospital, 2000 Frederiksberg, Denmark; ^3^Image Analysis Ltd, Leeds, BD17 7EZ, UK; ^4^Department of Rheumatology, University of Genoa, 16126 Genoa, Italy; ^5^Department of Rheumatology Hvidovre, Herlev Hospitals, 2730 Herlev, Denmark; ^6^Rheumatology Division, Kennedy Institute, Imperial College, London, SW7 2AZ, UK

## Abstract

Dynamic contrast-enhanced MRI in inflammatory arthritis, especially in conjunction with computer-aided analysis using appropriate dedicated software, seems to be a highly sensitive tool for monitoring the early inflammatory treatment response in patients with rheumatoid arthritis. This paper gives a review of the current knowledge of the emerging technique. The potential of the technique is demonstrated and discussed in the context of a case report following the early effect of an intra-articular steroid injection in a patient with rheumatoid arthritis flare in the knee.

## 1. Background

Imaging modalities aiming to identify perfusion characteristics in inflammatory joint disease are receiving increasing attention after results from a recent publication have shown that measures of perfusion detected with ultrasound Doppler in the wrist joints of rheumatoid arthritis patients with low disease activity scores (DAS28) had the highest predictive value of future erosive outcome [1] compared to both clinical measures and conventional contrast enhanced MRI. 

dynamic contrast-enhanced MRI (DCE-MRI) is such an imaging technique based on sequential acquisition of rapid MRI sequences before and during the infusion of a contrast agent. It can be used to evaluate synovial activity in patients with rheumatoid arthritis (RA) and has been shown to correlate closely to synovial vascularity and inflammation [2–4]. An enhancement curve is obtained, where the initial rate of enhancement and the resulting plateau and potential washout depends on the inflammatory vasodilation, neoangiogenesis, and perfusion. The early enhancement rate determined by DCE-MRI has shown to be more sensitive to change after intraarticular steroid injection [5] and has a closer relation to histological inflammatory activity than measures of the synovial volumes [4, 6], making DCE-MRI a promising tool for assessing the early inflammatory response to treatment, potentially even before volume changes, and thus changes in the semiquantitative synovitis score occur [7]. 

DCE-MRI has been tested on low-field [8] and high-field [4, 6] scanners and is capable of discriminating patients with clinically active disease from those in remission. 

Conventionally, DCE-MRI data is analysed using region of interest- (ROI-) based technique, where a small, few millimetre ROI is placed in the most enhancing part of the synovium, as perceived by an observer [8]. It has been shown that the size and position of ROI have a great impact on diagnostic accuracy and ROI misplacement by only a few millimetres might give a 20%–30% difference in the results [9]. Thus, ROI-based methods generate highly subjective and potentially unreliable results. Finally DCE-MRI data is influenced by micromovements of the imaged joint introducing artifactual enhancement, which results in large variation in the mean dynamic curves obtained by the ROI method [10]. 

These issues have been addressed by application of a new technique for analysis of dynamic data developed by Kubassova et al. [11, 12]. This approach is based on a fully automatic voxel- and model-based analysis technique with built-in movement correction, which improves signal to noise ratio up to 3-fold by taking out inter-scan patient motion artefacts. Application of this technique for analysis of dynamic data can solve most of the above-mentioned technical issues, making DCE-MRI a more robust and even more promising tool for assessing the early response of inflammation to treatment.

## 2. Objective

To use DCE-MRI data to monitor early changes in parameters of knee joint inflammation in a patient with a flair of RA following ultrasound-guided intra-articular injection of glucocorticoid (methylprednisolone acetate 40 mg/ml). The case will serve as an example of the technique and the changes seen will be discussed and explained in detail in order to give the reader a better understanding of the potential and pitfalls of using computer-aided analysis of DCE-MRI data. We hope that this paper could serve as an example of the potential of this methodology that can be further investigated in future larger studies. 

## 3. Case

### 3.1. Clinical Information

This 52-year-old lady was affected by seropositive RA diagnosed 13 years before. The patient had side effects with several DMARDs, including methotrexate and was treated with prednisolone, 5 mg daily. Supplementary injections of methylprednisolone were given occasionally in joints with acute flares; the last intra-articular injection was performed in a wrist joint 10 months before the present treatment. 

Clinical findings at baseline included a moderately swollen knee and slight-to-moderate joint pain with a 100 mm visual analogue scale of pain of 30 mm at rest and 50 mm on joint movement.

Joint aspiration yielded 25 cc of clouded synovial fluid, and after arthrocenthesis, 1.5 ml glucocorticoid methyprednisolone 40 mg/ml was injected in the lateral recess of the knee with almost complete resolution of symptoms within day 2 of injection and complete clinical remission at day 7. The effect lasted for 2 months. The patient had normal kidney function measured by serum creatinine and estimated glomerural filtration rate (e-GFR). 

### 3.2. Imaging

After informed and written consent, the patient had conventional static MRI as well as dynamic MRI performed on day 0, 1, 2, and 7 using a 0.2 T musculoskeletal extremity scanner (Esaote E-scan). The patient was examined in supine position with the knee positioned centrally in the receive-only cylindrical solenoid knee coil. The following pulse sequences were applied: gradient-echo scout, sagittal STIR (TR/TE/TI: 1310/24/85, fov/matrix: 200 × 170 mm/192 × 163, slice thickness 4 mm) and axial 3D T1 gradient echo (TR/TE: 38/16, fov/matrix: 180 × 180 × 100 mm/192 × 160 × 72, slice thickness 0.8 mm). After these images were acquired, an intravenous injection of 0.1 mmol/kg body weight Gadolinium-DTPA (Magnevist, Schering AG, Berlin, Germany) was administered over a period of 30 seconds. At the time of Gadolinium injection, 30 consecutive 5 mm axial gradient echo dynamic MRI (DCE-MRI) images (TR/TE 60/6, FOV/imaging matrix 160 × 160 mm/256 × 128) in three prepositioned planes were started and obtained every 10 second, covering the superior, medial, lateral, and posterior joint recesses in the knee. Image time was 300 seconds. Finally, the static axial 3D T1 gradient echo sequences were repeated. The acquisition time of each sequence ranged from 4 to 8 minutes, with one signal acquired. Total imaging time was 30 minutes.

### 3.3. Image Analysis

**Figure 1:**
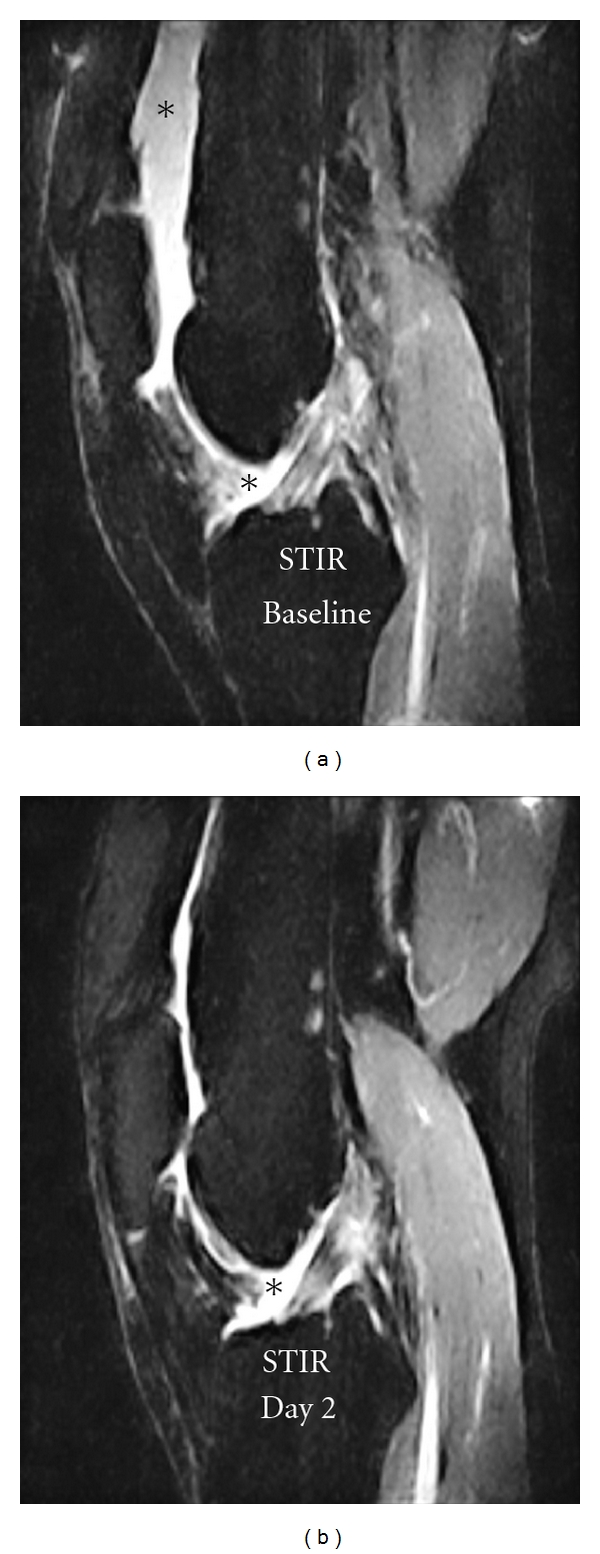
STIR images showing an evident signal decrease from the joint cavity between baseline and day two, corresponding to a reduced joint effusion. The effusion in the images is marked with an asterix (∗).

The conventional static imaging data was displayed using an AGFA PACS system (Figures 1 and 4(c)-4(d)). The STIR sequence (Figure 1) was used to evaluate the bone marrow and effusion. The pre- and post contrast 3D T1-w gradient echo images were used to evaluate synovitis using a previously published semiquantitative scores [7].

The dynamic enhancement pattern in the inflamed knee synovium was analyzed using the software Dynamika-RA (http://www.dynamika-ra.com/). Using this software, we reduced patient motion artefacts between the dynamic frames, which allowed reduction in artifactual enhancement, thus increasing the SNR by a factor of 2 (data not shown). The motion correction procedure took 3-4 minutes. 

**Figure 2:**
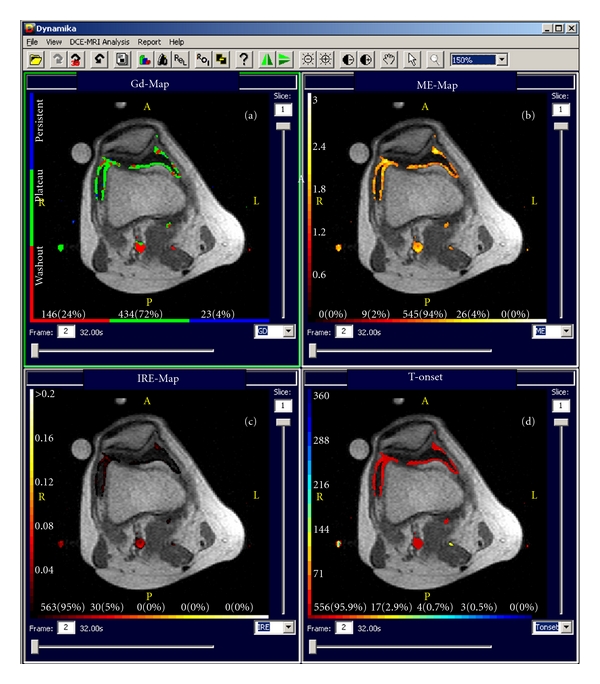
Parametric maps derived from the same DCE-MRI dataset reflecting the Gadolinium behaviour and distribution over time (Gd), maximum enhancement (ME), initial rate of enhancement (IRE), and time onset of enhancement (T-onset). The maps are derived from the baseline images of the case study representing a flair of moderate arthritis activity of the knee. Note the pulsation artefacts of the popliteal artery that give false “hot” points in the image related to a horizontal line of the artery.

Further, the data was analysed using the voxel-by-voxel-based approach, incorporated into the software, and the enhancement characteristics of each voxel was computationally mapped to one of 4 enhancement models [13]. Parametric maps of the Gadolinium uptake pattern (Gd), maximum enhancement (ME), initial rate of enhancement (IRE), and time of onset of enhancement (Figures 2(a)–2(d)) are automatically calculated, and the corresponding colours, representing the vessels perfusion and the synovial microcirculation, are superimposed on the gray scale dynamic precontrast T1-weighted image. The colours in the Gadolinium map reflect the behaviour of the Gadolinium over time, where voxels with no gadolinium uptake have no colour; voxels with persistent pattern of enhancement are shown in blue; voxels with plateau in green and voxels with washout pattern in red. In the ME and IRE maps, the most active contrast enhancing voxels are displayed in white to yellow colours, whereas tissues with less perfusion/inflammation are reddish (Figure 2) [13].

### 3.4. Understanding the Dynamic Enhancement Maps

The vertical colour bars or the *Y*-axis in the 4 enhancement maps displays the values of the chosen parameter (ME, IRE, Tonset, and Gd). The values are measured in each voxel, and then grouped into 10 equally spaced bins. These are displayed on the colour bar. ME shows the increase over a baseline in a particular voxel and ME is measured as a ratio between the baseline and the maximum enhancement of the enhancement model calculated by the software program. The IRE values show the increase in voxel intensity per second from time onset to maximum enhancement is reached. Tonset is measured in seconds and show the time in seconds, where the enhancement curve begins compared to the first baseline frame. Gd washout-red, plateau-green, and persistent-blue shows the pattern of enhancement in each particular voxel. 

The horizontal colour bar shows the number of voxels and their percentage of the total in ( ) for each statistic in the corresponding IMAP, for example, 1 (0.01%) or 248 (91%), and so forth. For more information, visit http://www.dynamika-ra.com/.

**Figure 3:**
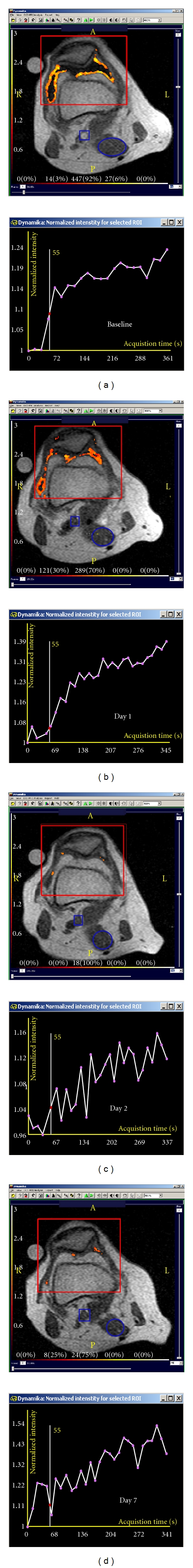
A rough ROI outlining the anterior part of the knee in the dynamic image guided by the ME maps and their corresponding dynamic curves from baseline through day 7 (a–d). Note the decrease of enhancing voxels in the parametric map over time as well as a simultaneous decrease of the slope in the corresponding dynamic enhancement curves. The *Y*-axis of the enhancement curves shows the intensity increase from baseline, and the *X*-axis shows the acquisition time in seconds.

**Figure 4:**
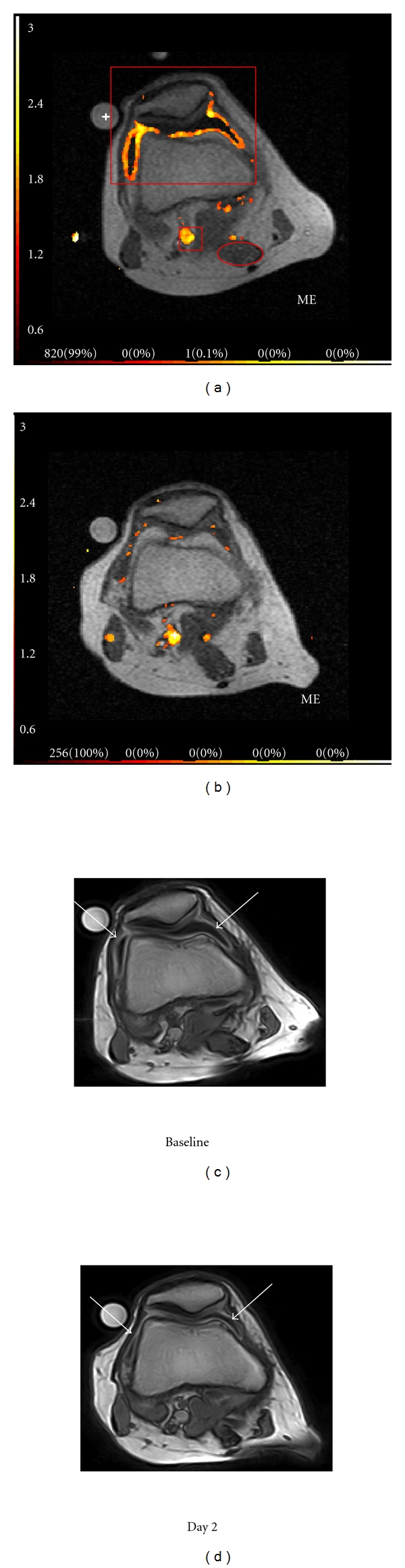
Parametric maps derived from DCE-MRI data maximum enhancement (ME) (a, b) and the corresponding static postcontrast 3D T1-w gradient echo images (c, d) from baseline and day 2. Arrows pointing at the enhancing synovial membrane in the post contrast images (c, d). Examples of the applied ROIs are shown in red in (a) representing the synovial ROI in front of the knee, the popliteal artery ROI behind the knee, and the muscle ROI (oval circle).

 After the movement correction and the fully automatic analysis of the knee joint was performed, several regions of interest (ROIs) were drawn (Figures 3 and 4): (1) a fast rough box ROI around the anterior part of the knee including the synovial membrane and excluding the major vessels; (2) a box ROI around the popliteal artery in the posterior part of the knee; (3) an oval ROI within the semimembraneous muscle (Figure 4(a)). An example of the maps of ME, IRE, and the corresponding static postcontrast 3D T1-w gradient echo images over time after intra-articular steroid injection are displayed in Figure 4. These maps serve as a guide to visually evaluate the effect of the steroid injection from baseline through day 7.

## 4. Results

The conventional STIR images (Figure 1) showed an evident signal decrease from the patients joint cavity between baseline and day two and a smaller signal reduction in the suprapatellar recess between day two and day 7. This signal reduction may be ascribed to significant decrease in joint effusion from a score of 2 to a score of 0 over time with maximum effect at day 7 [7]. Bone marrow oedema was not present. The corresponding postcontrast T1-weighted gradient echo images showed that the synovial enhancement (arrows Figure 4(c)-4(d)) was unchanged corresponding to a synovitis score of 1 [7] even though the volume of the enhancing synovium was visually reduced on day 7 (image not shown). 

## 5. DCE-MRI Data

### 5.1. Automatic Analysis

ME and IRE statistics, extracted from the dynamic data of this case and generated for the whole joint, showed no significant changes in the days following the steroid injection (Table 1). However, all Gadolium-related parameters, such as the total number of enhancing voxels (*N*-total), the number of voxels with wash-out (*N*-washout), and plateau (*N*-plateau ) pattern of enhancement, showed significant changes before and after treatment (Table 1). In contrast, an increase of IRE was noted at day 7.

### 5.2. ROI Analysis

We further outlined a rough ROI positioned to include the synovial membrane and to exclude the larger vessels especially behind the knee joint. There was no need to position ROI precisely, as the measurements were only done on the enhancing voxels inside the ROI (Figures 3 and 4). 

The IRE of the roughly outlined synovial ROI decreased from baseline values over the first two days by a factor of 4 and stayed in the low end at 1-week followup. The mean ME showed no significant reduction (Table 1). 

The dynamic curves and corresponding enhancement statistics from the vessel ROI including the popliteal artery remained relatively unchanged over time but showed a day to day variation (Table 1). 

In order to normalize the ROI data, we multiplied the sum of *N*-persistent and *N*-washout with the mean ME and mean IRE in all ROIs. This gave a much clearer treatment effect in the data revealing a significant reduction from baseline through day 7. We saw an effect even on day one with the most pronounced change between day one and two (Table 1). 

## 6. Discussion

When static post contrast T1 weighted MRI is used to monitor, the early inflammatory treatment response in patients with RA a change of up to 30% in enhancing volume is needed to imply a one step change in the inflammation score [14]; accordingly, this method is relatively insensitive to monitor the early treatment response upon anti inflammatory treatment. 

 In contrast, DCE-MRI seems to be highly sensitive to the early treatment response, but even though the methodology of DCE-MRI has been known for several years, previous studies have reported problems with reproducibility of results due to large variations in the ROI analysis [9]. The current case illustrates that DCE-MRI analysed using an appropriate computer software seems to be capable of detection and quantification of the very early treatment response and that the observed changes upon treatment in this case occur in parallel with changes in clinical symptoms. We have used a single case to illustrate the potential of the technique, but before final conclusions concerning broader utility of this method can be made, results from larger patient cohorts are warranted, and there are some pitfalls and technical challenges we need to understand as well. 

Fully automated data analysis of the whole joint revealed that the mean IRE and ME did not change significantly over time even though the number of enhancing voxels showed a dramatic decrease between day 1 and 2. The reason for this seems to be due to the confounding effect from the large vessels behind the knee, where the values of ME and IRE are the highest; thus, the enhancement changes in the synovial membrane are “shadowed” by the activity in the neighbouring vessels. On the other hand, making a rough ROI surrounding the synovial membrane, and thus removing confounding influence from the major blood vessels revealed a significant treatment response in the slope of the ROI curve that decreased by a factor of 4 between baseline and day 2 and remained in the same lower range at one-week follow-up. Based on these observations, we recommend to exclude the larger vessels from DCE-MRI analysis of the knee joint, which can be done by using a rough ROI and an appropriate software tool. 

The interpretation of changes in DCE-MRI following intra-articular steroid administration is also potentially confounded by the heterogeneity of treatment response across the whole synovial tissue mass. Thus, as expected, the ME did not change following treatment, since the remaining voxels demonstrating enhancement in the follow-up examinations reached approximately the same level as observed in pretreatment images. However, it took a longer time to achieve the plateau because of a lower steepness of the slope (Figure 3). In order to better interpret the changes in DCE-MRI images to reflect a true biological effect of treatment intervention, despite the regional variation in synovial responses which account for the lack of change in ME in the ROI as a whole, we normalized the data by multiplying the mean of ME and IRE by the number of voxels with plateau and washout pattern of enhancement. This permitted a much clearer statistical differentiation between the data acquired before and after the treatment. In our experience, all voxels, which reached the plateau or wash-out phase, seem to represent the areas with synovial and vessel perfusion, and we have chosen to use the sum of *N*-plateau and *N*-washout in this normalisation. In contrast, those tissues with a persistent pattern of enhancement (*N*-persistent) are often located in the skin area or are due to very fine movement artefacts that were not removed by the motion reduction algorithms. Whether this approach can be used in general needs to be clarified in larger studies. 

As the software uses a model-based enhancement classification, there is no need to apply a threshold, nor do we recommend to normalise the data to the enhancement characteristics of the vessels or the muscles, because there seem to be a relative large day to day variation in the ROI statistics. 

We have examined the current patient 4 times within a week to measure the effect of the steroid injection. This approach cannot be recommended for routine clinical use for many reasons, including the use of i.v Gadolinium, expensive and time-consuming MRI examinations, availability of MRI scanners for RA patients. The case should be seen as an example of the potential of the technique, but based on our results which have to be confirmed in larger studies, we speculate that there could be a benefit of using the technique to get a more objective idea of the early treatment effect of the more expensive biologic treatments, that is, within 2–4 weeks of treatment, which could lead to better patient care by reducing the time spend on an ineffective treatment and in the long run money could be saved on the health economy budget. 

This study has several limitations, as our findings are based on a case report, and we have used the knee joint to illustrate the potential of the technique, where ROI-based exclusion of the larger vessels is fairly easy. We cannot assume that our findings can be extrapolated to smaller and more complex joints like the wrist. The therapeutic intervention employed in this case was an intraarticular steroid injection known to have a potent anti-inflammatory activity, and we cannot assume that an observable treatment effect would be as pronounced and as rapid when using conventional DMARDS or even biologic therapies. 

## 7. Conclusion

In conclusion, DCE-MRI in conjunction with analysis using appropriate software seems to be a highly sensitive tool for monitoring the early inflammatory treatment response in patients with RA, as demonstrated by the assessment of a knee joint inflammation following an intra-articular steroid injection. The decrease in IRE and ME at day two follow-up in this case example, especially seen in the normalized data, corresponded to improvement in the patient's clinical symptoms. These findings have to be further tested in larger clinical trials on several joints to see whether the observed benefit in the current case using dynamic MRI may be used in general as a sensitive biomarker to track the early treatment response in patients starting potent anti-inflammatory treatments such as local/systemic steroid, and/or biologics.

## Figures and Tables

**Table 1:** 

Body part	Acq. Date	ROI name	Mean ME	Std. Dev. ME	Mean IRE	Std. Dev. IRE	*N*-persistent	*N*-plateau	*N*-washout	*N*-total	*N*-persistent + *N*-washout	ME normalized*	IRE normalized*
KNEE	Baseline	Fully automatic	1.59	0.23	0.010	0.01	45	609	185	839	794	1262	7.94
KNEE	Day 1	Fully automatic	1.59	0.37	0.011	0.02	57	590	313	960	903	1436	9.93
KNEE	Day 2	Fully automatic	1.63	0.28	0.015	0.02	28	119	132	279	251	410	3.77
KNEE	Day 7	Fully automatic	1.72	0.40	0.024	0.03	48	118	89	255	207	356	4.97

KNEE	Baseline	Synovium	1.59	0.23	0.008	0.01	29	531	85	645	616	979	4.93
KNEE	Day 1	Synovium	1.46	0.16	0.004	0.001	24	376	103	503	479	701	1.92
KNEE	Day 2	Synovium	1.43	0.09	0.002	0.001	16	39	23	78	62	88	0.12
KNEE	Day 7	Synovium	1.47	0.13	0.003	0.001	8	31	4	43	35	52	0.11

KNEE	Baseline	Vessel	1.79	0.19	0.038	0.02	0	10	65	75	75	134	2.85
KNEE	Day 1	Vessel	2.06	0.39	0.041	0.02	0	10	56	66	66	136	2.71
KNEE	Day 2	Vessel	2.00	0.24	0.037	0.01	0	10	51	56	56	112	2.47
KNEE	Day 7	Vessel	2.14	0.36	0.054	0.03	0	9	61	70	70	150	3.78

*Normalized values: sum of total number of voxels with plateau and washout information multiplied by the ME and IRE, respectively.
